# A digital companion for musicological scholarship: the Lohengrin TimeMachine

**DOI:** 10.1080/09298215.2025.2487100

**Published:** 2025-05-06

**Authors:** Laurence Dreyfus, David Lewis, Kevin Page

**Affiliations:** a Faculty of Music, University of Oxford, Oxford, UK; b Oxford e-Research Centre, University of Oxford, Oxford, UK

**Keywords:** Digital musicology, interdisciplinary collaboration, digital publishing, Wagner, multimedia, hypermedia

## Abstract

Musicology has long suffered from the difficulties of making its work accessible, or even comprehensible to a wider audience. We introduce a digital companion to music scholarship – in this case, an exploration of Wagner's early use of leading motifs in the opera *Lohengrin* – providing different ways to explore, see and hear the materials that are discussed in musicological research. That research is described in text and a video, each of which is incorporated into the companion as a springboard for further discovery. Novel visualisations include a ‘TimeMachine’ view, in which a user can flick through motif occurrences, quickly navigating through the musical transformations across the opera. Having introduced the scholarship and the companion, we discuss the collaborative process by which the application was conceived and built, including the practicalities of timing and incorporating external design expertise. We conclude by discussing the future of such ‘companions’ in musicological publication.

## 1. Introduction

In this article, we describe a digital publication that supports and enhances the communication of musicological scholarship. In our description of the work required to conceive, design and produce what we call a ‘digital companion’ to scholarship, we consider not only the technology and research required to produce it, but also the motivation, process and practical considerations involved. This was the product of a collaboration between individuals with differing areas of expertise, which is not uncommon in digital musicology. Such collaborations can require not only accepting different ways of thinking in others, but also accommodating them and adapting to them – this is true not only for the scholars in our case, but also for our graphic designer and videographers. We believe that the diversity of approaches inherent in interdisciplinary working is something to be celebrated and we do not conceal it here.

The work reported arises from conversations between the authors, facilitated by our participation in the *Transforming Musicology* project, itself an interdisciplinary collaboration. We begin with a musicologist's motivation, arising from a wish to use technology to communicate music scholarship more widely. This initiating perspective is summarised in Section 2, and grounds our work in a particular musicologist's approach and concerns. Section 3 provides a background to the hypertextual nature of our knowledge engineering work, while subsequent sections describe the companion itself (Section 4), and the process and practicalities of its implementation (Section 5). Finally, we consider what our digital companion might mean for future musicological publications.

## 2. A musicologist's perspective

This section describes the motivation for our work, from the perspective of a collaborating musicologist.

### 2.1. Provocation: limited public understanding of musicology

Unlike other humanistic disciplines, musicology has been plagued by a fundamental problem in reaching a broad reading public. For, in contrast to literature or art history in which scholars can simply quote a piece of text or supply an image to support an argument, musicology has traditionally required a high degree of musical literacy to grasp the sense of research which focusses on a discrete piece of music. Even a passing familiarity with musical notation that might stem from a reader's youthful instrumental lessons or choral experiences in church never ensures that they can hear in their head a musical example supplied in score within a musicological essay or monograph.^1^ Such a level of competence presupposes nothing less than years of tireless aural and keyboard training. Even the ability to play a musical instrument to a relatively high standard doesn't often lead to the competence to conjure at will both the sound and sense of a cited musical example.

A mathematician, say, who can decently cope with a Bach Prelude and Fugue at the piano might nurse a burning desire to understand the inner workings of Bach's music and so consults a specialised book on the topic only to find that even when she sits at the keyboard to play through the multitude of examples, the argument eludes her because of a lack of what Michael Polanyi (1958) called ‘tacit knowledge’. And even a trivial knowledge of what counts as tacit knowledge of musical grammar and affect – something unfortunately called ‘music theory’, as if it represents a science of inherent musical structures – isn't so straightforward when dealing with the history of European repertoires from the Middle Ages to the present day: this is the case because the syntax and semantics themselves were in a constant state of flux and were indeed made into the very object of musicological study. So we take it for granted that a musicologist will not be able to follow an advanced mathematical proof without years of specialised training in that discipline. Yet the music a particular mathematician wants to investigate is far from some remote and erudite science located in an academic ivory tower, but is actually an experience that plays a significant role in that mathematician's psychosocial existence and inner emotional world. For it is simply indisputable that for much of humanity, music wields an exceptional and extraordinary power in and over their lives.

Musicology's relative inaccessibility has extended even to colleagues in other arts disciplines who happen not to possess a sufficient level of musical literacy. These scholars are, for example, routinely warned away from interdisciplinary work that traffics in the nuts and bolts of art works, something which is not the case for musicologists needing to engage with literature, art history or the other performing arts. An art historian can readily illustrate a scholarly text with a reasonable facsimile of even large scale works along with a miniscule ‘detail’ extracted from, say, an oil painting. As a result, even researchers who lack formal training in painting or cultural history can immediately perceive what is both observed and analysed. In literature it's obviously no different, since even literature written in foreign languages can be furnished with translations. Within musicology, however, the assumption that only highly trained musicians would care to delve inside the workings of a piece of music has led to a perceived alienation from the discipline and has also encouraged a kind of mandarin style of discourse within musical analysis that makes it especially inhospitable and inapproachable. So whereas the sizeable role played by music in people's lives may be no less substantial than that of visual culture, the scholarly discourses that amplify and explain these experiences of art are not remotely comparable. It is no surprise, then, that the scale of worldwide sales of learned books on visual arts and literature simply dwarfs those treating music in any close detail. From the standpoint of musicology, this ought to be considered a dire state of affairs.^2^

The digital revolution and the resources of the internet have obviously made it easy to access sounding musical examples, but it is fair to say that musicology has been slow to exploit their potential both to illustrate the results of historical and analytical research as well as to allow for new forms of representation that accompany prose discourse. Exactly how to accomplish this, i.e. how to present compelling aural illustrations and develop new methods at home in a digital space, is a subject area that clearly requires deep thought.

Naturally this kind of collaboration has already led to some fascinating interactions between computer science and musicology, even if it's fair to say that the state of play is still in its infancy. Whereas corporate culture and the mass media have devoted seemingly limitless financial resources to harnessing digital tools to sell information and market products, the cost of replicating even a fraction of this expertise within the humanistic disciplines remains prohibitive, so that researchers face a plethora of obvious constraints. The arts subjects in general and music in particular are thereby left with a deplorable situation which the most advanced thought of the day fails to find a way to communicate its findings as effectively as it could, just as technological achievements in industry, natural science and media race past to leave it behind in the dust. It's in this light that our collaborative project on the Digital Companion to Leitmotifs in Wagner's Lohengrin sought to address this problem by taking some initial steps to show how much musicology has to gain from embracing the benefits of digital representation through new approaches to the display of relevant evidence.

In the case of treating a multimedia genre such as opera, a musicological argument needs to rely on a panoply of evidence. Not only is there an orchestral score which usually needs to be condensed into two or three staves of a so-called vocal (or piano-vocal) score, but in addition there is a poetic text along with stage directions which need to be quoted and translated as well as a range of possible images such as drawings and photographs of historically significant stage spaces and of the orchestral pit plus images of the relevant stage designs. Recordings – ideally even a range of recordings– need then to be linked and mapped onto each musical example in an musicological essay, for the act of performance naturally has a major impact on both sound and sense of the aesthetic experience, and practice has varied significantly since the opera's gestation.

So even when, say, a two-minute extract from an opera is cited to support a point in an argument, one wishes to provide the point's richest possible presentation and not confine it to the most ‘economic’ and slender rendering of the evidence. Rather than prejudge only what an author thinks a reader may need to follow the gist of the discourse, a bundle of contextual sources will always help flesh out a multi-perspectival ‘view’ (or audition) so that the reader becomes far more immersed in the entire historical and scholarly trajectory. In addition, it's possible – with the richest display of evidence and sources – to encourage the reader to spark new observations and perspectives which may go far beyond what might be thought traditionally necessary just to advance an individually plausible argument. This then leads to our notion of a digital companion which accompanies and exemplifies a specific piece of research and thinking, but also creates a resource that will aid future discoveries not yet envisaged.

### 2.2. Inspiration: Wagner and Leitmotif

Richard Wagner was of course not only a towering figure of 19^th^-century German opera but an artist of international repute and influence. Without him, much film music since the 20^th^ century would be unthinkable as would even – more perniciously! – easily recognisable jingles that play such a powerful role in mass-market advertising. Perhaps the most important reception of his works is what is called by most people leading motifs or leitmotifs – a term used for his stereotypical use of repeated musical figures identified with dramatic characters or actions.

A key, underexplored issue is that that the composer didn't like or accept the term ‘leitmotif’ (see, for example, passages in his collected writings, *Dichtungen und Schriften* Wagner, 1983, 9:334 and 8:364), so rather than ride roughshod over his refusal to accept this appellation, it's more interesting to ask why he did so, and what linguistic markers he did make use of to signal and explain for his varied repetitions and reformulations of musical snippets. With his first operatic successes in the early 1840s (Rienzi and the Flying Dutchman) and his last (Parsifal) in 1882, its also stands to reason that Wagner altered his ideas about his musical repetitions over the course of his compositional career. An important ramification of this observation is, that, rather than aspiring to propose a ‘theory’ of Wagnerian leitmotifs as many have attempted to do, it is far more worthwhile to chart what he said and did throughout his artistic life as regards his characteristic themes and motifs, that is, to pursue a fundamentally *historical* approach to this question.^3^

And to approach the historical question convincingly, one of course needs both analytical and documentary data: this is where an approach grounded in the digital humanities enters the scene. As a first step, we began to focus on one discrete Wagnerian work, and track and trace one motif throughout in exhaustive detail. Lohengrin (1850) was chosen as a key work in this regard as it's the last of Wagner's Romantic operas – the composer's designation!^4^ – and is positioned just before the conception of his mammoth 4-drama project called the Ring of the Nibelung, about which he theorised extensively in a published treatise entitled Opera and Drama (1851).

### 2.3. Conception: the Lohengrin TimeMachine

From this point of departure, we conceived the idea of the Lohengrin Time Machine, a device that would ultimately (ideally) contain all of Wagner's repeated and varied motifs in the opera. Why a Time Machine? The idea harked back to the name and look of Apple's eponymous back-up software first supplied in 2007 for the Macintosh operating system (MacOS), which created incremental backups of files which could be restored at a later date. With ‘snapshots’ of the Apple desktop shown to the user stacked atop one another – making use of Apple's Core animation API – one could, through the use of a sliding toggle, return in time to a date when a particular file was created or updated, and thereby restore the many stages of its reformulations. The design of the software imparted the sense that the time traveller, desirous of exploring the past, rushes easily through a seeming tunnel found in outer space, and is able to alight on any chosen date to uncover the state of the file data.

This then was the initial inspiration for the Lohengrin TimeMachine, in that the goal would be to produce software or a tablet-based app in which one could zoom to and fro in time so as to gain insights not only into how Wagner routinely modified, nuanced and reshaped his themes and motifs, but also to show in each case the poetic, theatrical and musical context of the occurrence. If there are some 19 iterations of the music called Forbidden Question that stretch across an opera that spans more than three hours of music, then our goal was first to load each ‘way station’ with musical, poetic, dramatic and analytic data so that a user can both see (in short score) and hear an example of the theme, supplied along with poetic text, stage directions in both German and English as well as scholarly commentary and even documentary evidence (such as relevant contemporary correspondence, diary entries and the like). In addition, even more ambitious than Apple's TimeMachine concept, was the ability to compare on one screen any two iterations of the same theme or motif side by side. Since we were able to devise a visual representation of the orchestration for each iteration of a motif, one could, for example, also observe and chart Wagner's constant changes in his choice of orchestral instruments over the course of the paradigmatic alterations of the theme, and see how these changes in colour and affect mapped onto the dramatic impulses sparked by poetic text and dramatic moment. The construction of the TimeMachine therefore led to the desideratum that subsequent users – whether humanities scholars or interested laity – would have access to a rich network of information which could spark new research or perspectives on Wagner, or on musical repetition and variation tout court.

A second feature of the TimeMachine was that it was especially suited as a documentary and analytical resource to accompany a scholarly historical essay – in this case – Dreyfus's new research on Lohengrin's motivic and thematic network, and Wagner's approach to motivic usage at this stage in his work. Rather than just supply notated and audio examples of the music and text, the reader following the musicological argument has the ability to act far more independently than a reader of a journal article: one doesn't just have to take what the author asserts as true to support a particular thesis or argument, but the reader of the essay can supplement and query individual points in the prose by zooming around the TimeMachine, enriching thereby immeasurably the experience of participating in and critically assessing a research output in historical musicology. That this extended utility of the TimeMachine is a real boon for the accessibility of musicological research and its potential outreach beyond academia is an exciting prospect that our project always wished to keep in mind.

## 3. Supporting and supplementing musicological publication with digital companions

Having described the musicological motivation and conception of the digital companion, in this section we turn to the motivation and approach from the perspective of technology and hypermedia. Central to the value of this digital companion is the combination of rich resources with an expert guide through them. The richer and more complex the information we publish, the more important the narrative structures become – particularly in the first steps of a companion user's interactions. These structures act as golden threads, leading us through the complexity, giving perspectives and priorities for future exploration. Thus, the companion supports and enriches scholarship; as a standalone edition, it has little value.

In order to make our companion function, the technology must satisfy some core capabilities. The resources that the companion calls upon, including the essay itself, arrive in various forms – formatted text, annotated score (vocal and orchestral), video, sound and image, and raw data. These multimedia resources are also interconnected by their context (for example, a recording and a score that present the same musical passage) and their interpretation (for example, two passages that are related by a musicologist's observation about them). To make the materials and the connections visible and allow them to be explored in an intuitive manner requires visualisations. Given a limited screen size for complex information structures, effective visual summary techniques are needed, but also dynamic, interactive approaches that remove the requirement to see all information at once.

The complex of interconnected resources we described can be most clearly described as a *hypertext*. Although this is a well-established concept – and HTML and related technologies have long been capable of supporting rich multimedia applications – hypertexts have relatively rarely been used to support scholarly musical narratives. When they have been used, they are often tailored applications with little reuse of code or data being possible^5^.

In library and museum contexts, web content is usually produced by generic content management systems, enriched with plug-ins for browsing digitised material from the organisation's collection. For example, the British Library system, SiteCore, powers sites such as *Discovering Literature* and *Discovering Music*. These are sites with an educational purpose, which are navigable by multiple paths and can be explored by topics (such as those required by UK schools' curricula). Such sites are hypertextual in nature, but the articles within them remain linear texts, even though augmented with media plugins where figures might be, including page turning and zooming of images, viewing of videos and so on.^6^

At the other end of the spectrum, less narrative-led, more exploratory digital library publishing systems such as Greenstone (Bainbridge et al., 2014) can also provide powerful ways of search and discovery in large digital collections. A rich, music-specific browsing experience has also been realised using Open Linked Data as part of the DoReMus project, bringing together media, and music-analytical and historical information (Lisena et al., 2017). Another framework using Linked Data, MELD (Music Encoding and Linked Data), was devised at the University of Oxford e-Research Centre, and has been used in a range of music-related multimedia applications. Previous uses of the MELD framework include an interactive composition (Kallionpää et al., 2017), a tool for annotating a score during a masterclass (Lewis et al., 2019), and a visualiser for comparing piano performances (Weigl et al., 2019); MELD provides the underlying implementation framework for the *Lohengrin TimeMachine* (Section 5.2).

What quickly becomes difficult in multimedia hypertexts is the tension between free exploration, where a user is given the tools to navigate complex materials in an unstructured, unconstrained way, and scholarly argument, where a musicologist navigates an explicit path through the data. The freedom that characterises the former may be well suited to exploratory research, but may overwhelm or confuse an inexperienced user. Our digital companion aims to balance the two, supplementing purely narrative paths with exploratory modes which give a user the freedom to find their own path, but informed by the scholarly narrative, and with the information presented explicitly shaped by the investigation it supports. We believe that this helps make the scholarship more readily accessible, and exploration of materials less daunting to a newcomer.

## 4. A tour of the *Lohengrin TimeMachine*, a digital companion

The *Lohengrin TimeMachine* digital companion is presented as a multimedia web application^7^ , optimised for tablet – specifically an iPad Pro – but fully functional on a desktop machine with mouse control. Using the application can be divided into two types of activity: (i) following a provided musicological narrative and (ii) exploring a digital companion to those narratives. In the sections that follow, we refer to these as narrative-led and exploratory modes.

### 4.1. Narrative-led modes

We provide two **modes** of narrative-led interaction – a textual **essay** and a **video**. Both narratives assess the way in which Wagner transforms motifs for dramatic purposes throughout the opera. These narratives discuss many musical elements, such as key region and orchestration, along with the structure of the motifs themselves, particularly the motif known as *Frageverbot*.

This motif is associated with Lohengrin's prohibition, banning his betrothed, Elsa, from asking about his identity or past. It is divided into two structures which can occur together or separately, an x-segment that introduces the ban (*‘Nie sollst du mich befragen’*, ‘Never shall you ask of me’) and a softer y-segment that alludes to Lohengrin's secret past (*‘woher ich kam der Fahrt,/ noch wie mein Nam’ und Art!'*, ‘whence I came, my name or my kind’) (these structures can be seen indicated and labelled in the score pane on the left in Figure 5 later in this article). The antagonist character Ortrud aims to defeat them both by persuading Elsa to break the prohibition, and the conflict between Ortrud's influence and Lohengrin's is played out in transformations of the motif in a way that the essays characterise as the ‘magical realm’ (Ortrud) and the ‘grail realm’.

#### The textual essay

**Figure 1.**
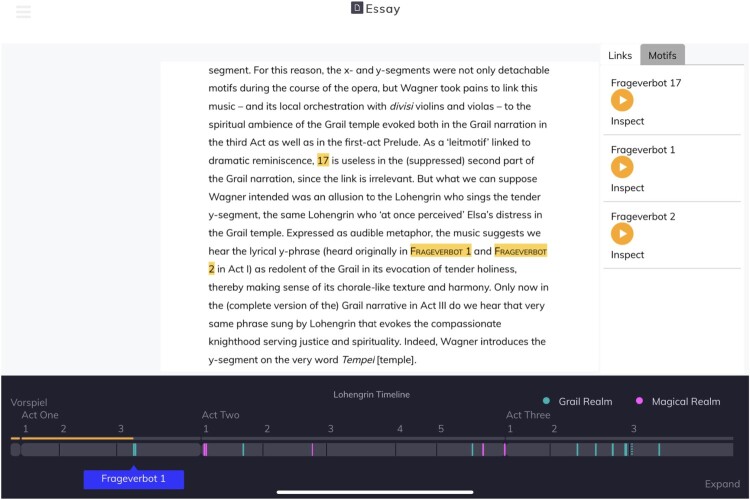
The musicological essay provides a traditional narrative that the reader can use as the basis for their exploration of the app. This mode shows the opera **timeline** (below) and links to other views (right). The Motifs tab on the right allows navigation within the essay text by motif iteration number, in a similar manner to a conventional index.

The text of the **essay** is presented to the user in the central pane of the screen (see Figure 1). Below, as on all screens in the application, is an automatically-generated **timeline**. The **timeline** is divided into acts and scenes, and each iteration of the *Frageverbot* motif is marked on the **timeline** as a vertical line, giving an overview of its distribution through the opera. The colour of line used reflects the dramatic character of that iteration – which of the two realms (‘grail’ or ‘magical’) dominates that occurrence. One motif iteration that was cut before the first performance is represented as a dashed line.

The application provides two dynamic elements to the user, both in the right-hand pane. An index of motif iterations provides jumping points for the essay itself, with short quotes from the prose helping the user choose where to jump to. An alternative tab in the pane (shown in Figure 1) responds to iterations mentioned in the visible text and provides recordings of them, along with navigable links into the relevant free-exploration part of the app.

The reader can thus either read the essay in a fully linear manner, with the added visual support of the **timeline** and audible support of the sidebar, or they can jump more freely around the essay itself, exploring the parts that discuss a particular iteration, or they can leave at any point to explore the application itself, returning to the point they left off.

#### The video essay

**Figure 2.**
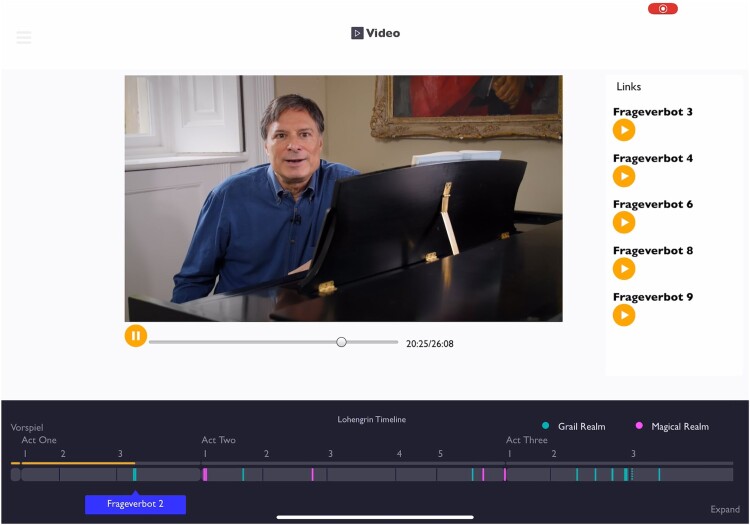
The video also provides an intuitive narrative-led introduction to the opera's use of motif and to the application itself. This mode shows the opera **timeline** (below) and links to other views (right), which appear as the video plays.

The **video** presents very similar supplemental elements to the user if they watch it from within the application – it is also available on YouTube. A similar pane layout (see Figure 2) puts the video in the centre with the **timeline** below. As the video is viewed, the right hand pane again provides audio clips from the opera and links into the more self-driven parts of the application.

Unlike the textual essay, the video also serves as a guide to the app, explicitly showing and referring to it, when the viewer is encouraged to click on links or listen to particular motifs. As for the essay, a viewer who followed an outward link will be able to continue watching, on their return, with the video player remembering the timestamp at which playback was paused.

### 4.2. Exploratory modes

**Figure 3.**
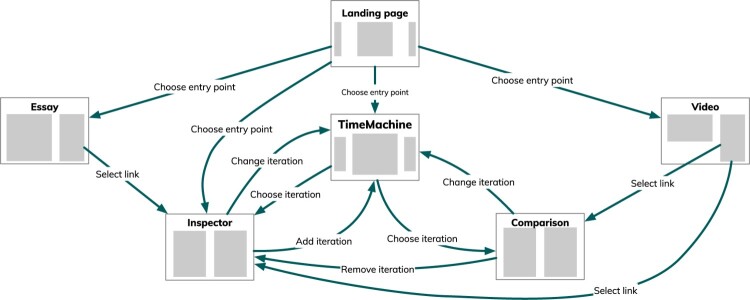
Diagram showing the main application pages and the flows between them. Links to exploratory modes can come from the **essay** or **video** modes, from the landing page, or from each other.

A user visiting the application website is presented with a landing page with links to the essay and video, but also offering two entry points into comparatively unguided exploration of the data – **inspector** and **TimeMachine** modes. These are two of the three exploratory modes in the application; the third, **comparison** mode, is less useful as a starting point. Figure 3 illustrates the implemented navigation paths a viewer can take to move between modes.

**Figure 4.**
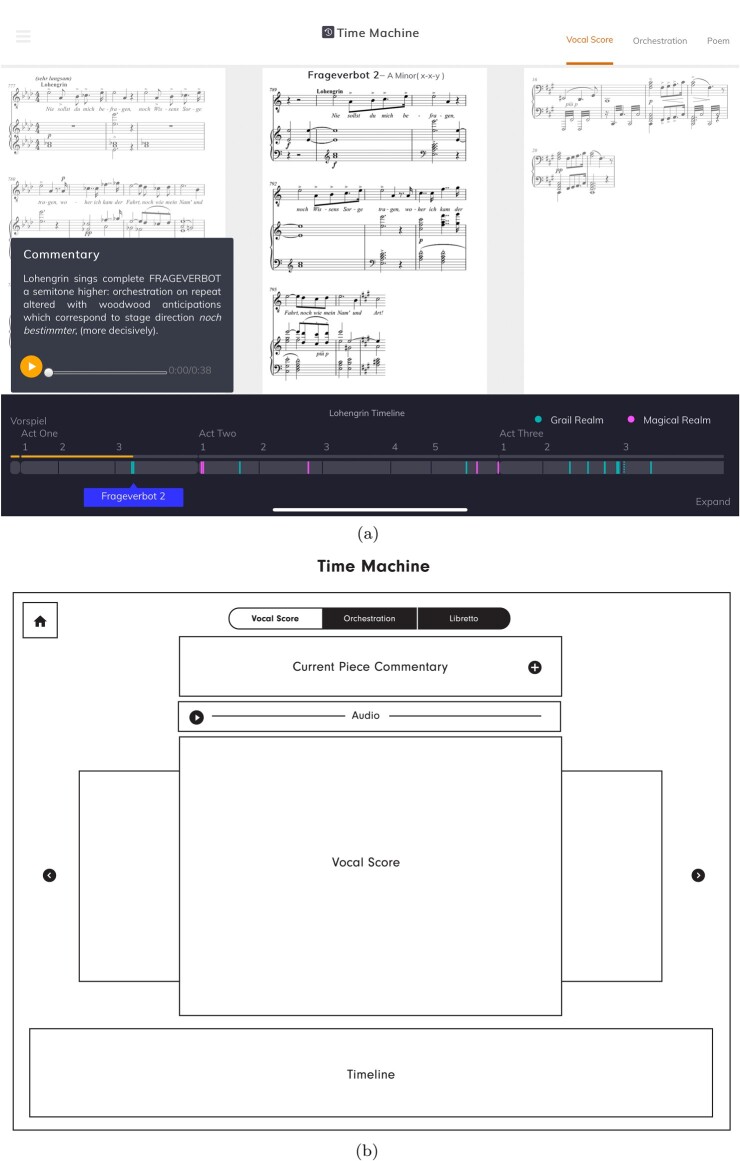
The **TimeMachine** mode, set to browse by vocal score. Flicking sideways scrolls through all the iterations in the order in which they appear in the opera, with the exact location highlighted in the **timeline** below. Browsing is also possible by orchestration and libretto (or ‘Poem’). Each iteration is accompanied by commentary text and a recorded extract. (a) In the app and (b) The designer's first low-fidelity wireframe.

The *Lohengrin* score is thousands of bars long (the second act alone has over two thousand bars), so it becomes crucial to support the reader in gaining an overview of change over time. The **timeline** introduced earlier provides a very high level abstracted overview of the opera, which also acts as an index – clicking on motifs in the **timeline** jumps the application to that motif. We provide a second, more detailed, overview in one of the exploratory modes – the **TimeMachine** mode (Figure 4). This mode summarises the sequence of motif iterations within the opera as an intuitive carousel-like interface, in which users can flick left or right to ‘time travel’ their way through the opera. The iterations in this mode can be visualised as score, libretto (or ‘poem’) or as an illustration of their orchestration (described below). Commentary on each iteration is displayed to the side.

**Figure 5.**
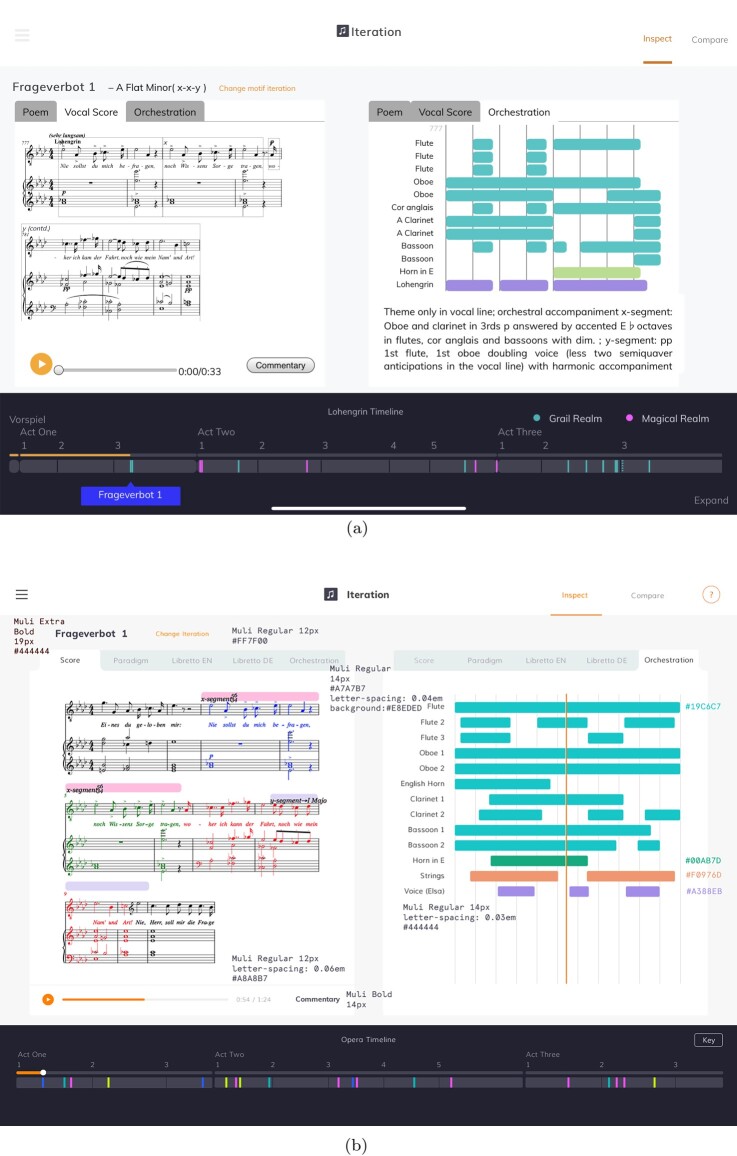
The **inspector** mode showing the vocal score and the orchestration visualisation side by side. The Poem, or libretto text, can also be viewed in German and English. The formal units identified in the score (for the *x* or *y* segments) can be selected, cueing playback to the beginning of that unit. (a) In the app and (b) The designer's high-fidelity wireframe.

Selecting an iteration in the **TimeMachine** mode, or following a link from the **video** or **essay**, will bring the user to the detailed **inspector** mode (Figure 5(a)). This mode offers the most information about the motif, including key and structure, vocal score with structural segments labelled, text underlay and stage markings (in German and English) and a visualisation of the orchestration of the extract. The layout is designed to make it easy to see these visualisations side by side, rather than one at a time.

**Figure 6.**
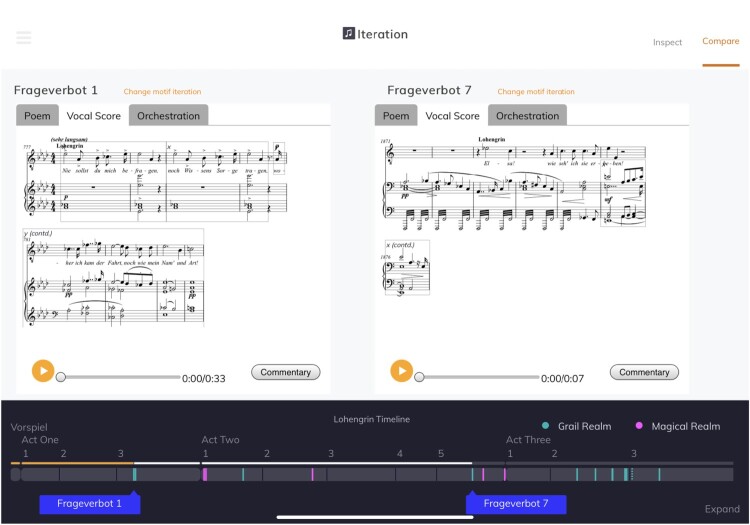
The **comparison** mode showing two iterations side by side.

From the **inspector** mode, the user can choose to **compare** motif iterations, bringing the **TimeMachine** mode back so that they can select another iteration. These are then displayed side by side in the **comparison** mode (Figure 6), which is functionally very similar to the **inspector** mode. The narrative-led modes can also link straight to a **comparison**, but in practice, only the **video** uses this capability.

Even if their combination and deployment is novel, most of the visualisations illustrated above, taken individually, draw on relatively well-established practice in both conventional and digital modes of presentation. One visualisation is less conventional, and warrants attention. It is an important part of the musicological narrative that it is not simply the notes in the motif iterations that supports the dramatic logic, but the timbre and orchestration. For each motif iteration, we link to encodings of both the vocal score and orchestral edition, but a Wagnerian orchestral score presents challenges within the constraints of the application. We do not target established academic musicologists as the primary audience for the application – and even those users might struggle with a full orchestral score, especially a Wagnerian one fitted into the dimensions of a tablet screen. To sidestep this problem, but still to support an understanding of the orchestration, we use a new, more abstract visualisation of an orchestral score, simplifying its visual complexity. In our orchestration pane, each instrument playing at a particular time is shown as a coloured ribbon, with the instrument's section of the orchestra providing the colour (seen in the right hand pane of Figure 5(a)). This visualisation, inspired in part by views in some music sequencer software, highlights differences in orchestration that may be invisible in a vocal score.

## 5. Implementing the digital companion

Creating a digital publication is a daunting, risky and expensive undertaking. In this section, we address the practical and technical aspects of the digital companion and then, in Section 6, we consider to what extent future publications might be made more achievable, and for a wider range of authors.

### 5.1. Practical considerations and the collaborative process

Conceiving, designing and creating the digital companion, along with web version of the textual and video essays, was a collaborative process throughout. It involved not just the authors of this article, but also academic colleagues, a designer, and a videographer. Nor did we start the process with a blank slate. Initial explorations into innovative ways of presenting scholarship around the topic of *Wagner and the Leitmotiv* had already begun within the Transforming Musicology project (2013–2017). It is this work that gave rise to the first version of Dreyfus's essay.

Nonetheless, with initial discussions within the team taking place in October 2016, and deployment late in August 2019, the whole process took almost 3 calendar years, supported initially by Transforming Musicology and then the Unlocking Musicology project (2018–2019). This timescale reflects not just the effort involved – no team member was employed full-time on the project – but the co-ordination of multiple collaborators and the time needed to develop and explore ideas.

Co-creation was at the core of our work, with ideas for the presentation and interaction of the application developed through intensive workshopping, using paper and whiteboard prototypes. Meanwhile, data modelling work began, with exchanges of spreadsheets of information (such as key, harmony and structure for each motif), along with Sibelius files of music examples. The music was converted to MEI and then the results corrected for use in the application.^8^ Based on the visual prototypes, the discussions and the data, development of software prototypes using the MELD framework could begin. Each software prototype was then circulated for comments from all team members, ensuring that the technical development remained aligned with the scholarly purpose.

At this point, we had the materials and the funding (through Unlocking Musicology) to take to web designers. However, we quickly found that our way of working – developing a prototype application before seeking a designer – is extremely unusual in the web development industry. Moreover, many web development companies aim to provide a full service for producing a web site. As a result, it proved difficult to find practitioners willing to provide interface and graphic design work for a system that, largely, already existed, and which had technical and resource constraints that were decided and fixed. It certainly seems desirable to have a designer involved as an active collaborator from the start of a process, but we note that it is not uncommon for the conception and exploration of new tools to be carried out before this is possible, and often before funding is available. Our experience is that, despite the difficulties in recruitment, a willing and collaborative designer will still prove extremely effective, even when joining at a relatively late stage and into an unfamiliar development approach.

**Figure 7.**
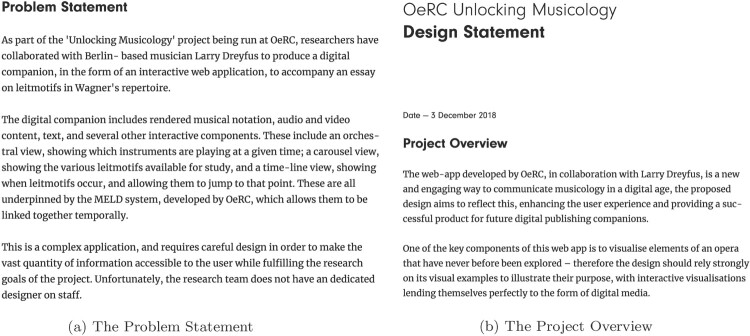
Samples of documents provided by the designer as part of the initial interaction (a) and the first phase of design (b). (a) The Problem Statement and (b) The Project Overview.

The chosen designer took briefing documents (formalised as a ‘Problem statement’, see extract in Figure 7(a)) and screenshots of the current state of the interface and its components. The designer met with the full team in November 2018 and produced a design document in December, including a prose ‘Project Overview’, and low-fidelity wireframe diagrams showing individual page designs and the flow between pages, clarifying structures and simplifying our layouts (see, for example, Figure 4(b) above). Once these had been discussed and any changes we needed implemented, the designer produced a high-fidelity wireframes, indicating the way each page should look (Figure 5(b) above). They also provided decorative images, fonts and details of sizes and colours for use in the redesigned companion. The modular design of the MELD system, along with the thoughtful and sensitive approach of the designer, meant that implementation of the new design proved quick and, largely, easy. This then formed the basis of a May 2019 meeting of the full team, to explore the new implementation and specify any changes, including ‘amends’ – changes for the designer to incorporate in his design document. These changes were enacted in the designs in June and in the code in July.

This left only scripting and recording the video – and, before that, recruiting a film-maker – and then adding the video to the application. This was completed in the following two months, with filming in Magdalen College, kindly made available by the Informator Choristarum, Mark Williams.

A typical process for developing tools in the digital humanities probably does not exist, but we hope that by providing this extra detail of our experience, we support those planning their own applications.

### 5.2. Software implementation

The TimeMachine is constructed as a MELD application – that is, it uses the MELD 2.0 framework (Weigl & Page, 2017). A MELD application can be seen as operating in two phases: graph building, and interaction. In its graph-building phase MELD traverses Linked Data graphs, successively requesting web-accessible Linked Data – with each document that is read potentially yielding links to new URLs to explore^9^. These URLs may lead to more Linked Data, from which the framework builds up a local knowledge graph within the browser, or they may lead to the multimedia resources that will contribute to the presentation of the application. The traversal engine thus allows apps to discover, select and filter relevant information. In its interactive publication phase, the MELD framework provides reusable components for creating and retrieving annotations, as well as for displaying and interacting with musical, textual, graphical and audio-visual materials.

MELD is written in JavaScript (as are its apps, including the *Lohengrin TimeMachine*). It uses the React framework, and operates over resources and data structured using Web standards. Linked Data for the application both uses and extends those structures expected by core MELD libraries, drawing on ontologies including the Music Ontology, Dublin Core, FRBR and, crucially, Web Annotations. FRBR is used here not only at the level of the complete work, but also to relate the abstract concept of, for example, the *Frageverbot* motif to the iterations of that motif that occur in the original score and, from those, to the editions and recordings that we include. This helps bridge the semantic gap between addressable resources on the one hand (represented by URLs or collections of URLs with fragment identifiers) and musical ideas that would be named, discussed and manipulated in a musicological investigation.^10^

For textual and musical materials, we benefit from the existence of stable XML-based standards for representing content in machine-readable ways: TEI^11^ and MEI^12^. In both cases, the use of XML means that, putting a document on the web at a particular URL also makes all the elements within the document available to be specified as URLs. More directly, any element with an xml:id can be directly specified within our linked data using a fragment identifier (# in the URL). In the *Lohengrin TimeMachine*, all textual content, including the essay, commentary and historical texts, is served as TEI, and rendered to the screen using CETEIcean^13^. Music notation is encoded as MEI, and either rendered using a MELD component that calls the Verovio toolkit^14^ (in the case of the vocal scores) or using our new orchestration viewer. The decision of which renderer to use is made dynamically, based on RDF indicating whether the score is for orchestra or for piano and voices. Both Verovio and CETEIcean preserve structures and xml:ids in their output where possible, allowing regions identified by the Linked Data as important to be located in the rendered output. Where Verovio (and the orchestration visualiser) outputs SVG images, CETEIcean creates HTML with custom tags. Both of these are placed within the page DOM and can be easily manipulated with JavaScript and styled using CSS.

The orchestration viewer is implemented as a reusable React component within the main MELD module (meld-clients-core) and generates an SVG image which is placed directly into the DOM. Ribbons are drawn live from an MEI encoding of an orchestral score, extracting the locations and durations of notes, and instrument names. Instrument labels are derived from those in the file, but can be overridden in the component configuration. This configuration also allows the definition or redefinition of orchestral sections – defaults are provided, but unusual instruments and singer names will usually be configured by the calling application. The component is capable of merging instruments that play together throughout an extract, but limited manual merging (such as for the first and second flutes) was preferred for the *Lohengrin TimeMachine* app.

All code and libraries are released as free and open source software, and can be freely re-used and re-deployed elsewhere. The MELD library may be found at https://github.com/oerc-music/meld-clients-core, while the *Lohengrin TimeMachine* itself is at https://github.com/oerc-music/ForbiddenQuestion. Other MELD apps are also available, and additional information may be found at meld.web.ox.ac.uk.

## 6. A future for digital companions

We believe that the *Lohengrin TimeMachine* illustrates an approach to musicological publishing that is new and powerful, and that much musicological research could be made more accessible and easier to follow through the use of comparable ‘digital companions’.

There has already been much work on ways of visualising aspects of musical structures and patterns and, although some of the visualisations and interface design is entirely new for the *Lohengrin TimeMachine*, it is the combination of different views of the music to support a coherent musicological investigation that gives it its power. We can use comprehension aids such as juxtaposing different musical elements, graphically summarising changes that happen over large time spans, and using sound examples and diagrams to make the most abstract aspects of score reading tangible and concrete.

But in making assertions, we should not ignore the practical cost. The development process of the companion was time consuming, and required an unusually close conversation between musicological and computer-technical expertise. We can conceive of two models for the further development of digital publications such as this. Firstly, future publications might follow a comparable process to ours, allocating similar time, money and expertise. Secondly, one might imagine a software environment for authoring new companions – just as editing tools exist for textual documents, music typesetting or creating websites.

This second option would be extremely attractive, allowing musicologists freedom to build exciting new publications on their own, and without yoking them to technologists. However, music research is less predictable than text typesetting. Although research in this area would be very welcome, it seems very unlikely that it would be possible to design software that provides generic, intuitive interfaces to allow researchers to express entirely new research in the clearest way possible, to a wide audience, since novel research will almost certainly require new modes of presentation. Perhaps a more realistic approach would sit between these two models.

### 6.1. Generalising the companion

As we touched upon earlier, the companion is built by combining software components using a library called MELD. Each time a MELD project builds a visualisation (such as the orchestration visualisation used in the companion), a decision is taken whether that functionality might be useful in other contexts; if so, the code is transferred to the core MELD library rather than remaining only in the particular application's source. In this way, future projects can build on visualisations built for other applications, only implementing new interactions as needed, and mostly – we hope – simply providing research-specific data and views on top of a modular, reusable library. Dad MELD not existed in some form at the start of our work, we would not have completed it within 3 years.

Just as our software components are modular and transferrable, so, crucially, is our data. If visualisations and interactions are to be re-used, it is extremely helpful for the data they build on to be structured in a way that is generic, powerful and likely to work in a variety of contexts. In our case, we use a combination of TEI, MEI and Linked Data standards such as the Web Annotation Ontology.

The use of general-purpose data structures presents another opportunity. While the most obvious and direct moment to enter the data on which a digital companion relies is during the process of developing the application, there remains the possibility of creating some of it even earlier. Within the later *Beethoven in the House* project, we have developed an application to support earlier stages of research, where a scholar can look at images or digital scores of the music they are studying and annotate points of musical correspondence, along with their own commentaries (Lewis et al., 2023). The data structures produced by this tool are compatible with the ones used by the companion. Although the researcher building a digital publication is unlikely to *exclusively* use information that they enter through the interface, the presence of such a resource could certainly reduce the complexity associated with publishing their scholarship in digitally-enhanced ways.

Other tools, such as those used by the FWF *Signature Sound Vienna* project also work with and produce MELD-compatible resources, expanding further the idea that any scholarly activity that takes place with these tools can short-cut, somewhat, parts of the development process (VanderHart et al., 2023). In the process of research using these tools, valuable records are produced of the intermediate stages of musicological research, often including materials that are valuable, but which don't fit comfortably within the narrative of a traditional academic paper.

We have given our focus in this discussion to static, one-off publications, supporting a single act of disseminating musical scholarship. This in itself is valuable, and we believe justifies the effort undertaken. Still, research is an incremental process, and digital publication could support this in several ways. The *TimeMachine* itself could be extended to illustrate *all* the motifs in *Lohengrin*, or to compare their treatment here with motivic development in later works. In both cases, further design and development work would be needed to maintain coherence, and more scholarly text would be needed to act as a guide within the extended companion, but the data and other materials that have been marshalled here could easily be re-used for such a purpose. And, since they are published online, with stable URLs, any team, located anywhere could build such an extension using our data.

### 6.2. Reflections on interdisciplinary collaboration

Re-use and re-purposing of digital materials on the web need not be limited to the published materials and analyses of earlier scholarship. The more the materials we marshal as evidence that can be published – in a digital form online in a way that allows free, open and direct referencing – the easier this becomes. Had the score or libretto of *Lohengrin* already been digitally published, our effort would have been reduced, and we would have had a lesser need to find the means to make our own raw materials available. Thus the development of rich, public digital editions and of the musicology that builds on them is best conceived as an ecosystem, where each participant benefits from the others in a virtuous cycle that makes it possible to share the excitements of music scholarship as widely and effectively as possible.

Beyond the technological work reported here is an enterprise building on research in multiple domains, where very little could be achieved without conversation, negotiation and mutual understanding. We sometimes speak as if such collaborations were a sign of the youth of our field or a disciplinary weakness, as if it should be incumbent on musicologists to become experts in all relevant digital technologies; instead, we reflect that collaboration amongst a diversity of views and voices enhances the result and is an important strength of the digital humanities. Meanwhile, the undertaking to communicate scholarship to a broader base is hard to evaluate – and we do not run visitor analytics on our companion – but at the time of writing, the accompanying video published on YouTube had attracted approximately 12,000 viewers, which must surely indicate some measure of success.

## Footnotes

1 Our point here is most acute for notated music, either transmitted in written documents or transcribed by a scholar for analysis or discourse. We appreciate that music need not be written down, but even in those cases, it remains at a level of abstraction that may present greater barriers than other arts. For discussion to go beyond lyric contents, a degree of musical knowledge and shared theoretical framework is usually required.

2 This reliance on musical training makes comprehension of this musicology vulnerable to changes to music education itself – both the broadly positive, such as the increased diversity of musics under study, and the wholly negative, such as the significant loss of music teaching and instrumental training in schools and the scope changes and closures of university music departments (Pace, 2023).

3 Many scholars have provided overviews of theories of leitmotif – this is a very large field – with more recent examples including Thorau (2009) and Deathridge (2008)

4 That is, Wagner consistently referred to most of his operas, in scores and elsewhere, up to and including *Lohengrin* as *Romantische Oper* (Rienzi was styled a *Große Tragische Oper*, and the abandoned *Das Liebesverbot* as a *komische Oper*. After this, Wagner started to use generic terms, which he made up himself, such as *Bühnenfestspiel*

5 For a Wagnerian example, see The Media Cafe's CD-ROM, *The Ring Disc* from 1997.

6 In October 2023, the British Library was the victim of a cyberattack by the hacker group *Rhysida*. As a result of this attack, all of their services were taken offline and the digital platforms used are in the process of review before services are restored. Although SiteCore itself will not be restored, the capabilities of its replacement are expected to be broadly similar.

7 Readers will find a link to the application itself at https://um.web.ox.ac.uk/lohengrin

8 Conversion from Sibelius was carried out via MusicXML, filtered through the MuseScore music typesetting package and finally converted using the Verovio toolkit. Recent developments that allow MuseScore to export MEI post-date our efforts on the companion and it is likely that they would have simplified our work.

9 This approach is often called ‘following your nose’ (see, for example, the relevant section of Dodds & Davis, 2011, rev. 2022).

10 This model for describing the relationships between thematically-related musical material has been developed and extended in subsequent work on shared material between musical arrangements, the *Music Annotation Ontology* reported in Lewis et al. (2022).

11 The Text Encoding Initiative, https://tei-c.org

12 The Music Encoding Initiative, https://music-encoding.org

13
https://github.com/TEIC/CETEIcean

14
https://verovio.org/
